# A commensal streptococcus hijacks a *Pseudomonas aeruginosa* exopolysaccharide to promote biofilm formation

**DOI:** 10.1371/journal.ppat.1006300

**Published:** 2017-04-27

**Authors:** Jessica A. Scoffield, Dingyu Duan, Fan Zhu, Hui Wu

**Affiliations:** 1 Department of Pediatric Dentistry, School of Dentistry, University of Alabama at Birmingham, Birmingham, Alabama, United States of America; 2 State Key Laboratory of Oral Diseases, Department of Periodontology, West China Hospital of Stomatology, Sichuan University, Chengdu, China; University of Maryland, UNITED STATES

## Abstract

*Pseudomonas aeruginosa* causes devastating chronic pulmonary infections in cystic fibrosis (CF) patients. Although the CF airway is inhabited by diverse species of microorganisms interlaced within a biofilm, many studies focus on the sole contribution of *P*. *aeruginosa* pathogenesis in CF morbidity. More recently, oral commensal streptococci have been identified as cohabitants of the CF lung, but few studies have explored the role these bacteria play within the CF biofilm. We examined the interaction between *P*. *aeruginosa* and oral commensal streptococci within a dual species biofilm. Here we report that the CF *P*. *aeruginosa* isolate, FRD1, enhances biofilm formation and colonization of *Drosophila melanogaster* by the oral commensal *Streptococcus parasanguinis*. Moreover, production of the *P*. *aeruginosa* exopolysaccharide, alginate, is required for the promotion of *S*. *parasanguinis* biofilm formation and colonization. However, *P*. *aeruginosa* is not promoted in the dual species biofilm. Furthermore, we show that the streptococcal adhesin, BapA1, mediates alginate-dependent enhancement of the *S*. *parasanguinis* biofilm *in vitro*, and BapA1 along with another adhesin, Fap1, are required for the *in vivo* colonization of *S*. *parasanguinis* in the presence of FRD1. Taken together, our study highlights a new association between streptococcal adhesins and *P*. *aeruginosa* alginate, and reveals a mechanism by which *S*. *parasanguinis* potentially colonizes the CF lung and interferes with the pathogenesis of *P*. *aeruginosa*.

## Author summary

The cystic fibrosis (CF) airway is a polymicrobial environment that is typically dominated by the destructive pathogen *Pseudomonas aeruginosa*. However, oral commensal streptococci are increasingly being recognized as relevant members of the CF polymicrobial biofilm and some studies have shown that these streptococci can potentially dampen *P*. *aeruginosa* virulence and positively influence disease outcomes. Unfortunately, knowledge of interspecies interactions in a mixed oral commensal streptococcal and *P*. *aeruginosa* biofilm is limited. Hence, we utilized a dual species biofilm model to study *P*. *aeruginosa* and oral streptococcal biofilms. Our findings demonstrate that an oral commensal exploits the production of a premier *P*. *aeruginosa* exopolysaccharide to enhance biofilm formation while simultaneously restricting biofilm formation by *P*. *aeruginosa*. Our data also show that streptococcal adhesins play a critical role in promoting exopolysaccharide-dependent biofilm formation by the commensal. The novel findings presented here provide insight into how oral commensals may infiltrate the CF polymicrobial biofilm and inhibit *P*. *aeruginosa* pathogenesis.

## Introduction

Biofilms are a consortia of bacteria that frequently dwell on medical devices, as well as environmental and biological surfaces. Often, biofilms are comprised of diverse bacterial species that participate in synergistic interactions and contribute to recalcitrant infections. In addition, bacteria living within a biofilm are typically more resistant to antimicrobials and have the ability to evade clearance by the host immune response [1–3]. Since biofilm-associated bacteria are recalcitrant to various treatments, biofilm formation often contributes to the development of chronic infections. Pulmonary infections in cystic fibrosis (CF) patients are a prominent example of a chronic infection that is characterized by the presence of multiple species of bacteria colonizing the CF airway and the ability of *Pseudomonas aeruginosa* to establish decade-long infections in the lung [4, 5].

The most common microbes detected during early infection of the CF lung include *Burkholderia cepacia*, *Staphylococcus aureus*, *Haemophilus influenzae*, and *Streptococcus pneumoniae* [5, 6]. Co-infections from two or more bacterial species are common in the CF lung. However, most CF patients are eventually colonized with recalcitrant variants of *P*. *aeruginosa*. *P*. *aeruginosa* is the most clinically important pathogen in CF patients because it causes lung deterioration and mortality [7]. More recently, studies have shown that the presence of oral commensal streptococci in the CF airway correlates with improved lung function [8]. Oral commensal streptococci are often recognized as primary colonizers of the tooth surface because they provide a platform for late colonizers to form complex biofilms [9]. Current evidence suggests that oral commensal streptococci have the ability to disseminate to body sites that are distant to oral cavity, such as cases with infective endocarditis and CF pulmonary infections [8, 10]. However, the interactions between oral streptococci with distant pathogens like *P*. *aeruginosa* are unknown. Most bacterial interaction studies involving *P*. *aeruginosa* have mainly focused on characterizing microorganisms that have been historically found to be co-colonized with *P*. *aeruginosa* in the CF lung. Since oral commensals are now emerging as a clinically relevant player in the CF environment, more studies are examining how these bacteria modulate *P*. *aeruginosa* virulence and influence CF infections.

Our laboratory previously reported that H_2_O_2_-producing oral commensal streptococci can inhibit *P*. *aeruginosa* in a nitrite-dependent manner [11, 12], which represents a protective mechanism by which commensal streptococci may improve the lung function of CF patients. However, factors that contribute to the incorporation of oral commensal streptococci into the CF polymicrobial biofilm are not fully understood. In this study, we characterized the interaction between *P*. *aeruginosa* and oral commensal streptococci in a dual species biofilm. We demonstrate that mucoid *P*. *aeruginosa* promotes biofilm formation by *Streptococcus parasanguinis in vitro* and colonization *in vivo* through interactions with surface exposed streptococcal adhesins. This study reports a previously unknown experimental association between the commensal *S*. *parasanguinis* and *P*. *aeruginosa*.

## Results

### The mucoid *P*. *aeruginosa* isolate, FRD1, promotes *S*. *parasanguinis* biofilm formation

**Fig 1:**
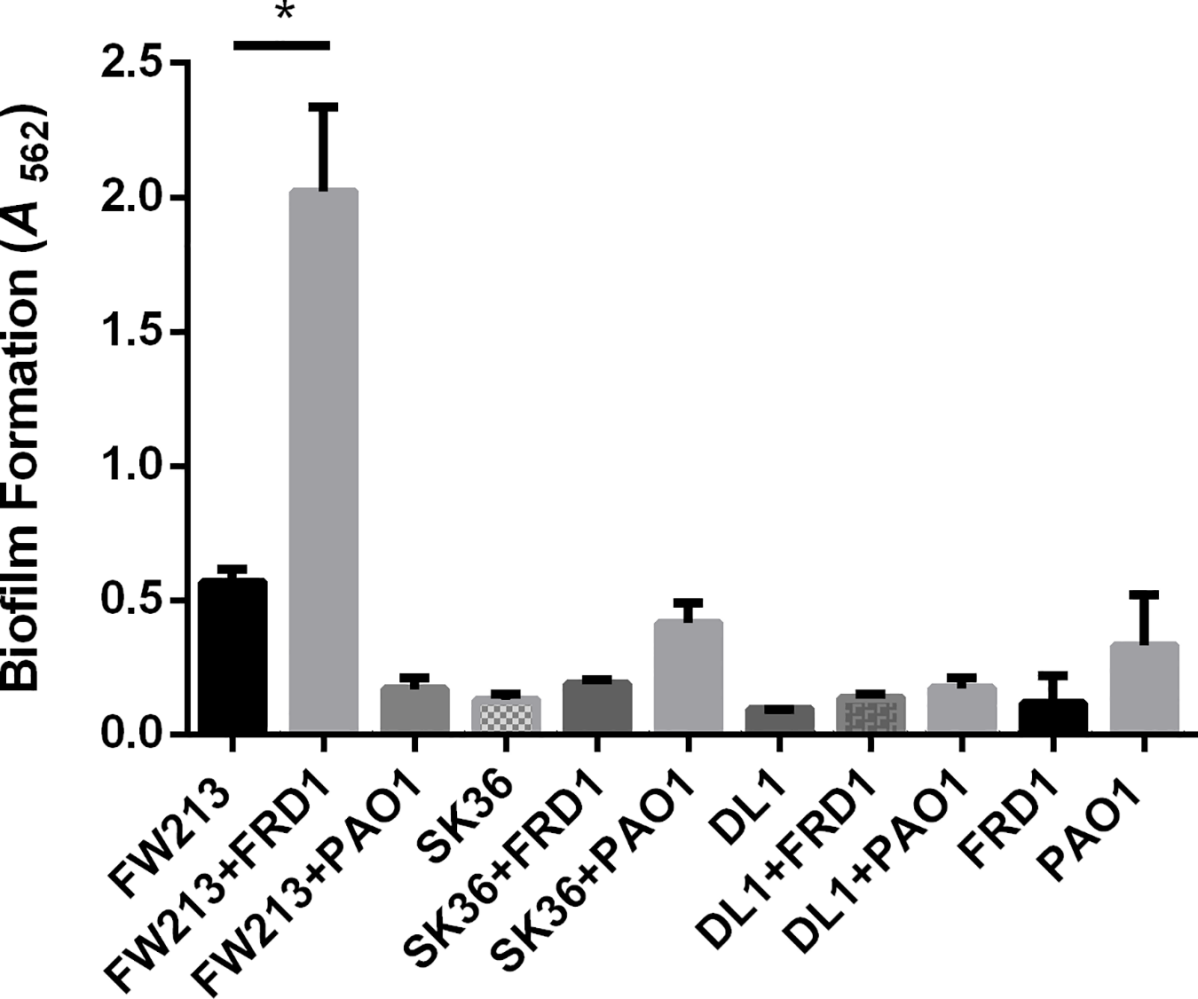
Survey of oral streptococci and *P*. *aeruginosa* dual species biofilms. A. 16 hour dual-species biofilm formation by *P*. *aeruginosa* strains FRD1 and PAO1 with oral streptococci, *S*. *parasanguinis* FW213, *S*. *sanguinis* SK36, and *S*. *gordonii* DL1. *P<0.05 (Student’s *t*-test).

**Fig 2:**
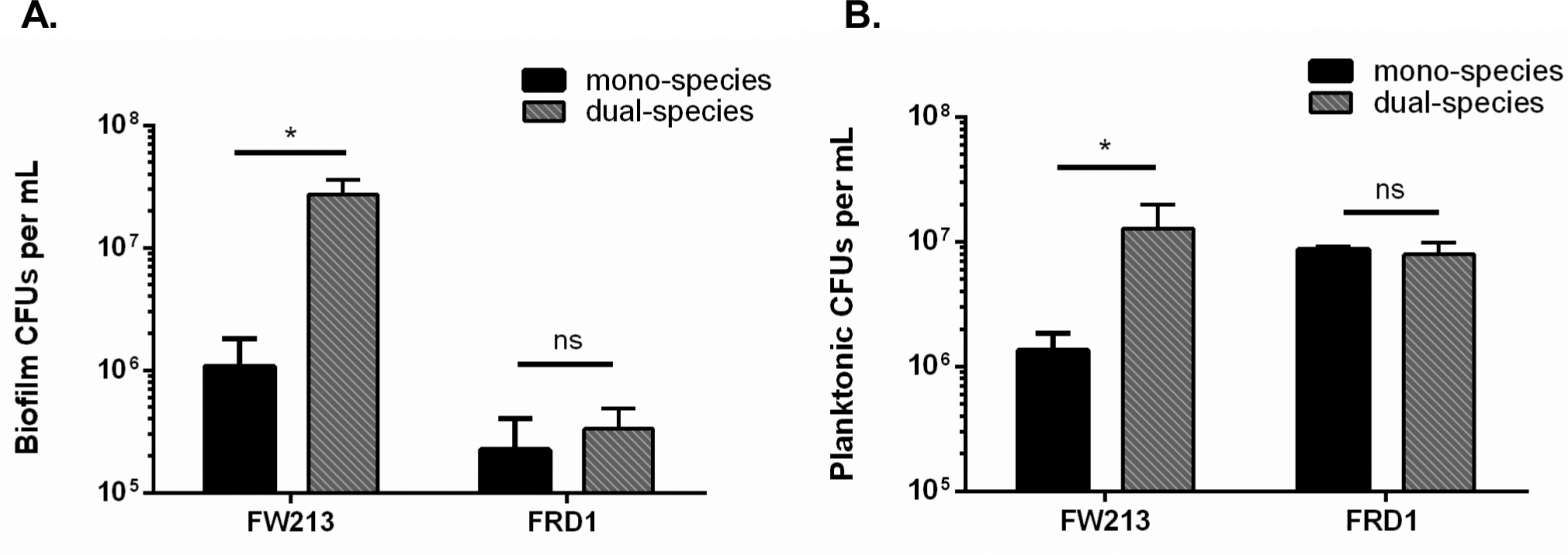
The *P*. *aeruginosa* chronic CF isolate, FRD1, promotes biofilm formation and planktonic cell growth by *S*. *parasanguinis* FW213. A. Quantification of FW213 and FRD1 cells in a six hour single and dual-species biofilm. B. Quantification of FW213 and FRD1 planktonic cells in single and dual species cultures. Data are representative of three experiments performed in triplicate. *P<0.05 (Student’s *t*-test).

In an effort to define the nature of two species biofilms containing *P*. *aeruginosa* and oral commensal streptococci, we quantified biofilm biomass when either *P*. *aeruginosa* isolates FRD1 (chronic CF isolate) or PAO1 (wound isolate) were co-cultured for sixteen hours with the following oral commensals: *S*. *parasanguinis* (FW213), *S*. *sanguinis* (SK36), and *S*. *gordonii* (DL1) using a crystal violet assay. Strikingly, there was a ~3 fold increase in biomass in the FRD1 and FW213 two-species biofilm compared to the mono-species biofilms of FW213 or FRD1 (Fig 1). However, co-culture with FRD1 did not increase biofilm formation with SK36 or DL1 (Fig 1). In addition, the two species biofilms with PAO1 and all of the oral streptococci resulted in no significant increase in biofilm biomass compared to the mono-species biofilms (Fig 1). To quantify the contribution of FW213 and FRD1 in the dual species biofilm, we measured colony forming units (CFUs) using a six hour biofilm. The presence of FRD1 promoted the number of FW213 biofilm cells by more than one log compared to the single FW213 biofilm, however, FW213 did not promote the biofilm of FRD1 (Fig 2A). Furthermore, FRD1 enhanced the growth of FW213 planktonic cells, whereas the number of FRD1 planktonic cells was not increased when co-cultured with FW213 (Fig 2B). Since FRD1 and not PAO1 can enhance biofilm formation by FW213, this suggests that FRD1 likely harbors some unique characteristics that are critical for the observed phenotype.

### Alginate production by *P*. *aeruginosa* is required for enhanced biofilm formation by *S*. *parasanguinis*

**Fig 3:**
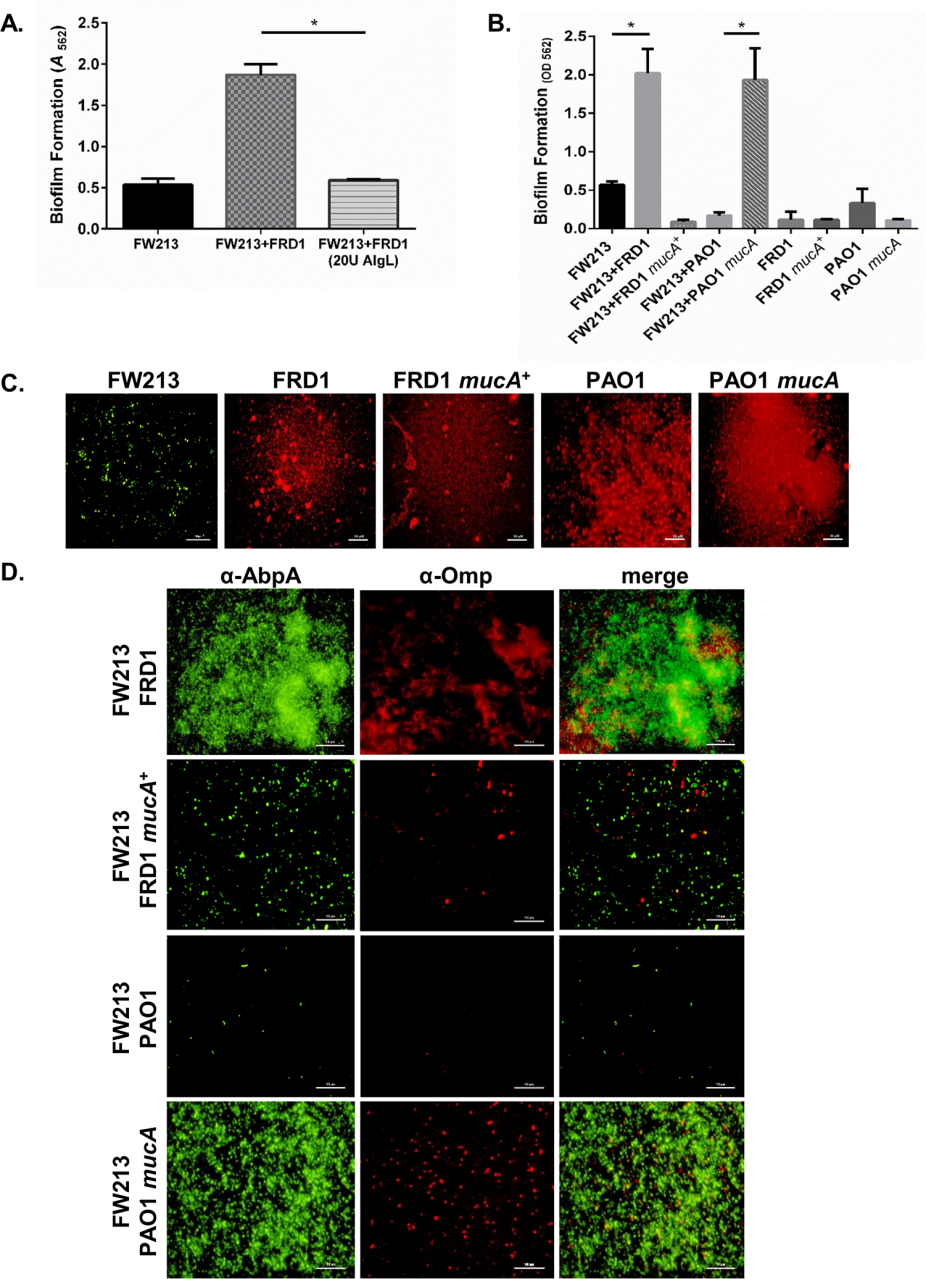
*P*. *aeruginosa* alginate producers enhance biofilm formation by FW213. A. Effect of alginate lyase on the formation of FW213 and mucoid FRD1 16-h biofilms. B. Dual species biofilm formation of FW213 with FRD1, FRD1 *mucA*^+^, PAO1, and PAO1 *mucA*. C. and D. Immunofluorescence microscopy images of single and dual species biofilms of *P*. *aeruginosa* strains and *S*. *parasanguinis* FW213 at 40x magnification. FW213 was probed with an α-AbpA polyclonal antibody and stained with a goat anti-rabbit Alexa Fluor 488 secondary antibody. *P*. *aeruginosa* was probed with an α-Omp monoclonal antibody and stained with a goat anti-mouse Alexa Fluor 594 secondary antibody. Scale bar: 50 μM. Data are representative of three experiments performed in triplicate. *P<0.05 (Student’s *t*-test).

One prominent phenotypic difference between FRD1 and PAO1 is the mucoid colony morphology exhibited by FRD1. The mucoid phenotype is common in *P*. *aeruginosa* strains isolated from CF sputum [13]. Mucoidy is due to the overproduction of the exopolysaccharide alginate, which is caused by the loss of the anti-sigma factor *mucA* in FRD1 [13, 14]. Due to this major difference between the FRD1 and PAO1 strain, we hypothesized that the production of alginate may contribute to the dramatic increase in FW213 biofilm formation in the FRD1 and FW213 dual species biofilm. To this end, we tested whether the addition of alginate lyase, an enzyme that cleaves the β-1, 4 linkage of the alginate mannuronic and guluronic acid copolymer [15], could abolish the increase in FW213 biofilm by FRD1. FRD1 was unable to promote the biofilm of FW213 in the presence of alginate lyase (Fig 3A). It should be noted that alginate lyase has no effect on the bacterial growth of both FW213 and FRD1 (S1 Fig). To further confirm the contribution of alginate, we evaluated a non-mucoid FRD1 strain (FRD1 *mucA*^+^) complemented with a wild-type copy of *mucA* from PAO1, and a mucoid PAO1 strain (PAO1 *mucA*) with a mutation in *mucA*, for their ability to form dual species biofilms with FW213. Alginate producers in both the PAO1 and FRD1 backgrounds increased biofilm formation with FW213, whereas alginate non-producers in both backgrounds did not increase the dual species biofilm (Fig 3B). In addition, FRD1 mutants defective in *algD* and *algT*, which are required for alginate biosynthesis, did not enhance the FW213 biofilm (S2 Fig). These data demonstrate the importance of alginate in the dual species biofilm.

To directly examine the relative contribution of each bacterium within the dual species biofilm, we performed immunofluorescence microscopy studies to visualize FW213 and *P*. *aeruginosa* using amylase binding protein A (AbpA) and outer membrane protein (Omp) antibodies, respectively. Fluorescence imaging of the biofilms revealed that FW213 comprised the majority of the dual species biofilm with either *P*. *aeruginosa* FRD1 or PAO1 *mucA* (mucoid/alginate-positive) (Fig 3C and 3D). In addition, quantification of alginate production in the dual species biofilms demonstrated that FRD1 and PAO1 *mucA* produce comparable levels of alginate to that of a single species biofilm (S3 Fig). However, FRD1 *mucA*^*+*^ and PAO1, which do not produce alginate (S3 Fig), failed to promote biofilm formation by FW213 (Fig 3C and 3D). Interestingly, dual species biofilms of FW213 with FRD1 *mucA*^*+*^ or PAO1 were considerably reduced compared to the single species FW213, PAO1, and FRD1 *mucA*^*+*^ biofilms (Fig 3C and 3D). The reduction in biomass in the dual species biofilms with *S*. *parasanguinis* and *P*. *aeruginosa* non-mucoid strains cannot be attributed to a reduction in planktonic cell viability. Neither PAO1, FRD1 *mucA*^*+*^ or FW213 planktonic cells were inhibited when co-cultured together (S4 Fig). In fact, similar to FRD1, the presence of both mucoid and non-mucoid *P*. *aeruginosa* stimulated FW213 growth (S4 Fig). The ability of PAO1 and FW213 to coexist planktonically suggests that mechanisms exist that prevent non-mucoid *P*. *aeruginosa* and *S*. *parasanguinis* from committing to the biofilm mode of growth in the dual species model. Moreover, analysis of additional *P*. *aeruginosa* CF clinical isolates demonstrated that while mucoid and non-mucoid isolates can promote the dual species biofilm with FW213, the biofilm increase is more significant in mucoid clinical isolates compared to non-mucoid clinical isolates (S5 Fig).

### *S*. *parasanguinis* is spatially co-localized with alginate in the dual-species biofilm

**Fig 4:**
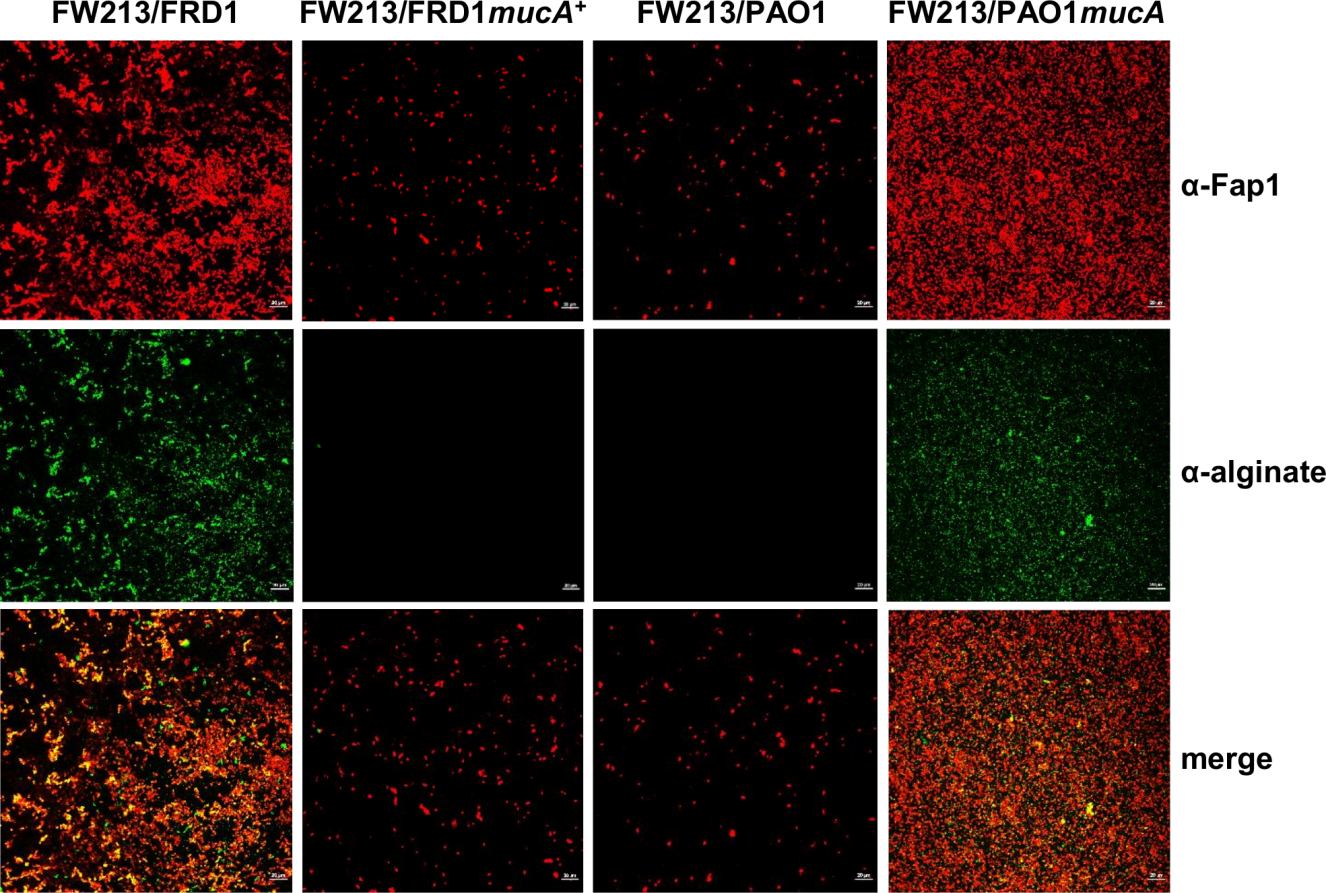
Co-localization of alginate and FW213 in the dual species biofilm. Confocal laser scanning microscopy (CLSM) images of FW213 and alginate in a dual species biofilm with FRD1, FRD1 *mucA*^+^, PAO1 and PAO1 *mucA* at 40X magnification. FW213 was probed with an α-Fap1 monoclonal antibody and stained with a goat anti-mouse Alexa Fluor 594 secondary antibody. Alginate was probed with an α-alginate polyclonal antibody and stained with a goat anti-rabbit Alexa Fluor 488 secondary antibody. Scale bar: 20 μM.

**Fig 5:**
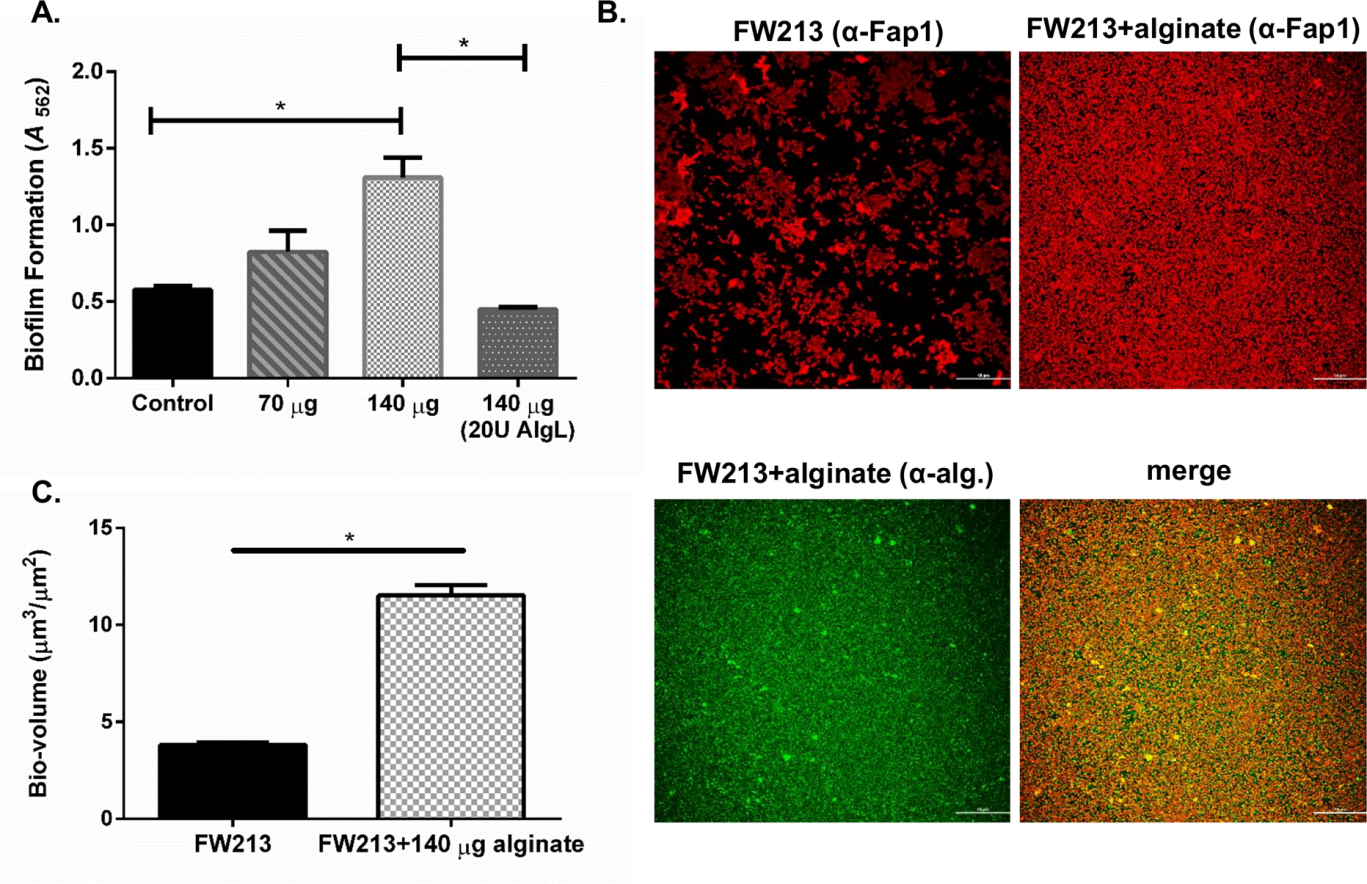
Purified *P*. *aeruginosa* alginate promotes FW213 biofilm formation. A. Biofilm formation by FW213 is increased by purified alginate and inhibited by alginate lyase dose-dependently. B. CLSM of FW213 control and FW213 with alginate (140 μg/milliliter) at 40x magnification. FW213 was probed with an α-Fap1 monoclonal antibody and stained with a goat anti-mouse Alexa Fluor 594 secondary antibody. Alginate was probed with an α-alginate polyclonal antibody and stained with a goat anti-rabbit Alexa Fluor 488 secondary antibody. C. Bio-volume analysis of biofilm depicted in ‘B’ using NIS Elements imaging software. Scale bar: 50 μM. Data are representative of three experiments performed in triplicate. *P<0.05 (Student’s *t*-test).

Fluorescence microscopy images illustrated that although the biofilm of FW213 is increased by *P*. *aeruginosa* alginate producers, FW213 is not spatially co-localized with *P*. *aeruginosa* cells, particularly since there are fewer *P*. *aeruginosa* in the dual species biofilms compared to single species (Fig 3C and 3D). These results indicate that *S*. *parasanguinis* possibly utilizes alginate produced by *P*. *aeruginosa* as a biofilm matrix component. To determine if this is indeed the case, we probed the two species biofilms using antibodies specific for alginate and the *S*. *parasanguinis* surface protein, Fap1. Confocal laser scanning microscopy (CLSM) imaging of these biofilms determined that the distribution of FW213 cells (red) directly overlapped with the distribution of alginate (green) in the biofilm, which is illustrated in the merged images (orange) (Fig 4). Pearson’s correlation coefficient and Mander’s overlap coefficient, which quantify the degree of co-localization between two fluorophores [16] (FW213/red and alginate/green), demonstrates a strong correlation between the overlap of FW213 cells and alginate (Table 1). Furthermore, purified alginate from FRD1 enhanced a single species FW213 biofilm in a dose-dependent manner, and this increase was abolished by alginate lyase (Fig 5A). CLSM of the FW213 biofilm in the presence of purified alginate also demonstrated the co-localization of alginate with FW213 cells, suggesting that alginate can directly influence the FW213 biofilm (Fig 5B). Quantitative analysis of these CLSM images further confirmed the increase in FW213 biomass with the addition of alginate (Fig 5C). Taken together, these data suggest that *S*. *parasanguinis* exploits the production of alginate by *P*. *aeruginosa* while simultaneously limiting the incorporation of *P*. *aeruginosa* into the dual species biofilm.

**Table 1:** Quantification of FW213/alginate colocalization in dual biofilms.

Sample	Pearson’s coefficient	Mander’s overlap
**FRD1/FW213**	0.611501*	0.696953*
**FRD1*mucA***^**+**^**/FW213**	0.206963	0.207955
**PAO1/FW213**	0.288194	0.287092
**PAO1*mucA*/FW213**	0.659952*	0.638586*

Coefficients > 0.8 indicate very strong correlation, 0.6–0.8 strong correlation, 0.59–0.4 moderate correlation, and < 0.4 is a weak correlation.

*Correlation is significant at the level of *P*<0.05.

### Sortase A anchored BapA1 is required for enhanced biofilm formation of *S*. *parasanguinis* by FRD1 *in vitro*

**Fig 6:**
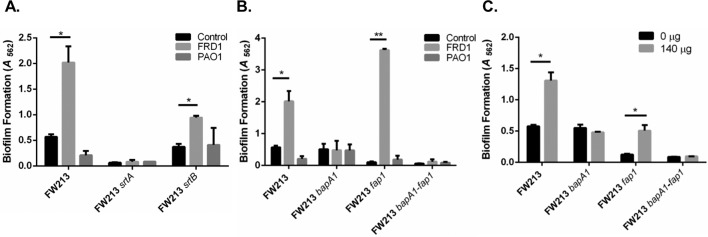
Sortase A anchored-BapA1 is required for the promotion of FW213 biofilm by alginate. A. Dual species biofilms by either FRD1 or PAO1 with the FW213 sortase A or B mutant. B. Dual species biofilms by either FRD1 or PAO1 with wild-type FW213 or FW213 *bapA1*, *fap1*, and *bapA1-fap1* mutants. C. Single species biofilms of FW213, *bapA1*, *fap1*, and *bapA1-fap1* mutants with purified alginate. Data are representative of three experiments performed in triplicate. *P<0.05 and **P<0.005 (*t*-test).

Next, we wanted to determine which biofilm-related factors of FW213 mediate the interaction between *S*. *parasanguinis* and alginate. Cell surface proteins anchored by sortase enzymes play a major role in modulating biofilm formation by oral streptococci [10,17,18]. We tested if a mutation in two distinct sortases (sortase A and B) would impair enhanced biofilm formation by *S*. *parasanguinis* in the presence of FRD1. A defect in sortase A abolished a mono-species *S*. *parasanguinis* biofilm and also inhibited the ability of FRD1 to promote the dual species biofilm (Fig 6A). As expected, the non-mucoid strain PAO1, had no effect on the sortase A deficient biofilm of *S*. *parasanguinis* (Fig 6A). In contrast, the biofilm of the sortase B mutant of *S*. *parasanguinis* was still enhanced by FRD1, but displayed a ~50% decrease in biomass compared to the wild-type FW213 and FRD1 dual species biofilm (Fig 6A). Amylase binding protein A (AbpA) is currently the only known surface protein anchored by sortase B in *S*. *parasanguinis* [17]. In an effort to explain the reduced biofilm by the FW213 sortase B mutant and FRD1 mixed biofilm, we co-cultured FRD1 and PAO1 with the FW213 *abpA* mutant. Similar to the results of the sortase B mutant, the *abpA* mutant was enhanced by FRD1, but to a lesser extent (two-fold) compared to wild-type FW213 (S6 Fig). This result suggests that loss of *abpA* likely explains the results of the sortase B mutant dual species biofilm with FRD1. As expected, there was no difference between the sortase B or *abpA* mutant mono-species biofilm and dual species biofilm with PAO1 (Fig 6A and S6 Fig). These data suggest AbpA plays a role in the promotion of the dual species biofilm, however, due to the complete abolishment of the FRD1 and sortase A mutant dual species biofilm, we turned our attention to sortase A controlled factors.

Fimbria-associated adhesion (Fap1) and BapA1 are two well-characterized sortase A-controlled surface adhesins that are involved in biofilm formation by *S*. *parasanguinis* [19, 20]. We tested whether a mutation in either *bapA1* or *fap1* would alter the phenotype of the two species biofilm with *S*. *parasanguinis* and *P*. *aeruginosa*. Compared to the wild-type FW213 dual species biofilms, the *bapA1* mutant and FRD1 dual species biofilm was not promoted (Fig 6B). As expected, the *bapA1* mutant and PAO1 dual species biofilm was also not promoted (Fig 6B). The dual species biofilm with the *fap1* mutant, which is deficient in biofilm formation compared to wild-type FW213, was considerably enhanced approximately 35-fold by the addition of FRD1 (Fig 6B). Furthermore, the addition of purified alginate enhanced the biofilm of the *fap1* mutant, but not the *bapA1* mutant (Fig 6C). Lastly, the *bapA1*/*fap1* double mutant biofilm was not increased by *P*. *aeruginosa* or purified alginate (Fig 6B and 6C). Overall, these data suggest that the streptococcal surface adhesin, BapA1, is necessary for enhanced biofilm formation by *S*. *parasanguinis* in the presence of alginate *in vitro*.

### *P*. *aeruginosa* isolate FRD1 Promotes the colonization of *S*. *parasanguinis* in *Drosophila melanogaster*

**Fig 7:**
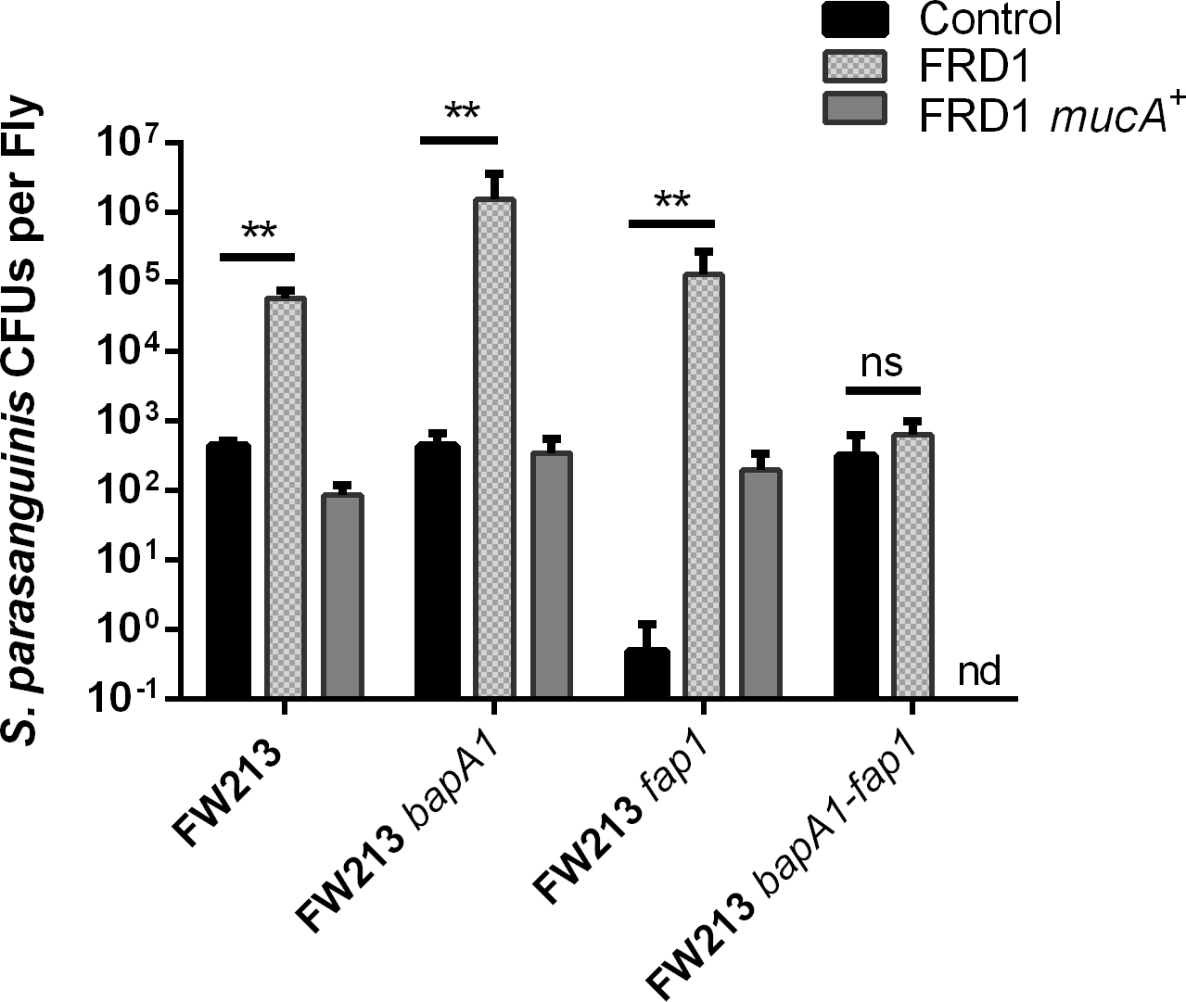
FRD1 promotes colonization of *S*. *parasanguinis* in *Drosophila melanogaster*. 24-hour colonization of *Drosophila* with single or co-infection with *S*. *parasanguinis* and *P*. *aeruginosa* using the following strains: FW213, *bapA1*, *fap1*, *bapA1-fap1*, FRD1, and FRD1 *mucA*^+^. ns = not significant. nd = not detected. Data are representative of three experiments performed in triplicate. **P<0.005 (*t*-test).

Thus far, our *in vitro* data suggest that a mucoid *P*. *aeruginosa* alginate producer, such as FRD1, can promote biofilm formation by *S*. *parasanguinis* FW213. As a result, we evaluated the influence that FRD1 (mucoid) and FRD1 *mucA*^+^ (non-mucoid) have on the colonization of *S*. *parasanguinis* using the *Drosophila melanogaster in vivo* model. Furthermore, we tested whether BapA1 or Fap1 mediate the colonization of *S*. *parasanguinis* in the presence of *P*. *aeruginosa*. Co-infection of *Drosophila* with FRD1 resulted in a ~2 log increase in the number of FW213 cells recovered compared to a single FW213 infection (Figs 7 and S7). However, FW213 did not promote the colonization of FRD1 compared to a single FRD1 infection (Figs 7 and S7). The non-mucoid FRD1 *mucA*^*+*^ strain did not promote FW213 colonization of *Drosophila*, however, the presence of FW213 reduced the colonization of FRD1 *mucA*^*+*^ (Figs 7 and S7). Surprisingly, co-infection with FRD1 enhanced the colonization of the FW213 *bapA1* mutant by ~3 log compared to the single *bapA1* infection (Fig 7). This *in vivo* data is inconsistent with the *in vitro* finding that FRD1 does not increase the FW213 *bapA1* mutant biofilm. FRD1 colonization was also increased by ~3 log during co-infection with the *bapA1* mutant compared to a single FRD1 infection or co-infection with wild-type FW213 (S7 Fig). Similarly, the FW213 *fap1* mutant and *bapA1-fap1* double mutant promoted FRD1 colonization (S7 Fig). In addition, non-mucoid FRD1 *mucA*^+^ did not promote colonization of the *bapA1* mutant *in vivo* and the *bapA1* mutant did not promote colonization of FRD1 *mucA*^+^ (Figs 7 and S7). The FW213 *fap1* mutant, which was defective for colonization of *Drosophila* by more than ~3 log compared to a wild-type FW213 single infection, was increased by ~5 log during co-infection with FRD1. Conversely, there was a ~3 log decrease in colonization of the *fap1* mutant when co-infected with FRD1 *mucA*^*+*^ in comparison to wild-type FRD1. Even though the FW213 *bapA1*-*fap1* double mutant was able to colonize *Drosophila*, FRD1 did not enhance colonization of the double mutant. In addition, FRD1 *mucA*^*+*^ completely inhibited the colonization of the double mutant (Fig 7). Alginate expression (*algD*) was maintained in *Drosophila* single and co-infections with mucoid FRD1 (S8 Fig). These results suggest that FRD1, an alginate producer, can promote *S*. *parasanguinis* colonization *in vivo*, whereas a non-alginate producer (FRD1 *mucA*^*+*^) cannot promote colonization. Furthermore, these data demonstrate that the presence of only one of the streptococcal surface adhesins, BapA1 or Fap1, is sufficient for enhanced *S*. *parasanguinis* colonization *in vivo*.

## Discussion

The CF airway is a polymicrobial environment that is typically dominated by the major pathogen, *P*. *aeruginosa*. The impact of a *P*. *aeruginosa* chronic infection in the CF lung has been extensively studied due to its association with lung dysfunction and mortality [21]. However, *P*. *aeruginosa* is not the sole colonizer of the CF airway, but exists among diverse bacterial species dwelling within a biofilm [6,22, 23]. Therefore, studies that examine interactions among bacteria found cohabiting the lung are important for understanding bacterial virulence mechanisms and CF pathogenesis.

Oral commensal streptococci are an emerging [8,23, 24], but understudied group of bacteria detected in the airways of some CF patients. Moreover, the presence of oral streptococci in the CF lung has been associated with improved lung function [8], however, no studies have closely examined the interaction between oral commensal streptococci and *P*. *aeruginosa* within a biofilm. In this study, we demonstrate that alginate production by *P*. *aeruginosa* promotes biofilm formation and colonization of *Drosophila melanogaster* by the oral commensal *S*. *parasanguinis*. Additionally, biofilm formation by the chronic mucoid *P*. *aeruginosa* CF isolate, FRD1, is not enhanced in the dual species biofilm. Furthermore, we find that the *S*. *parasanguinis* biofilm adhesin, BapA1, is required for the promotion of biofilm formation by *P*. *aeruginosa in vitro*, but is dispensable in the *in vivo* model.

The findings of our study are significant for two important reasons. First, exploitation of the mucoid phenotype to promote colonization of commensal streptococci could potentially interfere with *P*. *aeruginosa* pathogenesis during CF pulmonary infections. The conversion of *P*. *aeruginosa* from the non-mucoid to mucoid (alginate production) phenotype is a hallmark of a chronic *P*. *aeruginosa* infection and correlates with a decline in lung function in CF patients [25]. Mucoidy contributes to the formation of biofilms by *P*. *aeruginosa*, prevents the penetration of antibiotics, and inhibits phagocytosis and the activation of complement [1]. It has been previously shown that the occurrence of oral streptococci in the CF lung correlates with improved lung function (8). We previously demonstrated that oral commensal streptococci inhibit *P*. *aeruginosa* in a nitrite-dependent manner and the mucoid isolate, FRD1, is more sensitive to the inhibitory effects compared to the non-mucoid isolate PAO1 [11, 12]. Based on our previous and current findings, the incorporation of *S*. *parasanguinis* into the CF airway, which may be orchestrated by alginate production, could serve as an effective strategy to limit the impact of chronic infections caused by mucoid *P*. *aeruginosa* isolates. Secondly, this study highlights a novel association between a *P*. *aeruginosa* exopolysaccharide and a streptococcal adhesin that could potentially explain a mechanism of colonization by oral streptococci in the CF lung, which to date, has not been reported.

Bacterial adhesins, like BapA1 and Fap1, may facilitate the integration of oral streptococci into the CF pulmonary biofilm. BapA1 has previously been shown to be important for adhesion by *S*. *parasanguinis* [19] and may play a critical role for incorporation of this bacterium into the CF airway. In our study, BapA1 was required for the *in vitro* promotion of FW213 biofilm by FRD1. In addition, FRD1 enhanced the FW213 *fap1* mutant biofilm more than the wild-type FW213 biofilm. Functionally, BapA1 is involved in assembling short fibrils on the cell surface that can be masked by the long Fap1 fimbriae [19]. Therefore, we suspect that loss of Fap1 results in more surface-exposed BapA1, and as a result, the association between BapA1 and alginate is magnified *in vitro*. In contrast, the presence of either the BapA1 or Fap1 adhesin was sufficient to promote the colonization of FW213 by FRD1 in the *Drosophila in vivo* colonization model. Only deletion of both *bapA1* and *fap1* completely abolished the enhanced colonization of FW213 by *P*. *aeruginosa* in this model. It is unclear why BapA1 is not required for the enhancement of FW213 by FRD1 *in vivo*. One possibility is that within polymicrobial biofilms, BapA1 and Fap1 exhibit functional redundancy *in vivo* and not *in vitro*, and as a result, the presence of either adhesin is sufficient for alginate-mediated colonization of *S*. *parasanguinis* in *Drosophila*. Microbial adhesins often display divergent functions *in vitro* compared to *in vivo*. For example, a mutation in the major *Candida albicans* adhesin, Als3, results in defective biofilm formation *in vitro*, but not in a rat venous catheter model [26], suggesting that other factors can compensate for the loss of Als3 under *in vivo* conditions. Interestingly, deletion of Als1, another *C*. *albicans* adhesin with sequence similarity to Als3, forms a biofilm *in vivo*, but deletion of both Als1 and Als3 alleles fail to form biofilms in the *in vivo* catheter model [27], suggesting that Als1 and Als3 have overlapping roles *in vivo*. Likewise, we argue that BapA1 and Fap1 may function similarly in the context of an *in vivo* polymicrobial infection since the presence of either adhesin facilitates enhanced colonization of *S*. *parasanguinis* by mucoid *P*. *aeruginosa*, but the absence of both abolishes the observed phenotype.

Amongst oral bacteria and fungi, adhesins and pili are typically involved in direct cell-cell interactions that control bacterial coaggregation and biofilm formation. Als3 not only controls biofilm formation by *C*. *albicans*, but facilitates interkingdom biofilm development and attachment to the oral commensal, *Streptococcus gordonii* [28–30]. Furthermore, the *S*. *gordonii* adhesin, SspB, is directly involved in interacting with the Mfa1 surface protein of *Porphyromonas gingivalis* to promote *P*. *gingivalis* biofilm formation, and also mediates binding to an *Actinomyces oris*-derived polysaccharide [31–33]. However, unlike previous studies that demonstrate adhesin-specific interactions between bacteria that are generally specific to the oral cavity, our study highlights a unique association between an oral bacterial adhesin and a polysaccharide produced by a traditionally non-oral bacterium. Whether Fap1 and BapA1 possess lectin activity that binds to the alginate polysaccharide awaits further investigation.

*P*. *aeruginosa* produces three polysaccharides (alginate, Pel, and Psl) that participate in different stages of biofilm development [34]. Numerous studies have examined the role of these exopolysaccharides in *P*. *aeruginosa* mono-species biofilm formation [34, 35], however, few have evaluated the contribution of these exopolysaccharides in polymicrobial biofilms. One study has shown that alginate and Psl play a larger role in mediating the integration of *P*. *aeruginosa* into a mixed species biofilm rather than promoting the presence of another bacterium [36]. Moreover, the influence of these exopolysaccharides in mixed species biofilm development is highly dependent on post-transcriptional regulation of each polysaccharide, since the biosynthetic pathways of each polysaccharide compete for common sugar precursors [37]. High production of one polysaccharide may limit the production of another. For example, PAO1 produces a negligible amount of alginate and higher quantities of Pel and Psl, whereas FRD1 produces copious amounts of alginate and less Pel and Psl [34,37, 38]. As a result, it is unlikely that Pel and Psl contribute to the enhanced biofilm of FW213, which is supported by the inability of three different non-mucoid FRD1 strains (*algD* and *algT* mutants and *mucA* complemented) and the ability of the alginate overproducing strain, PAO1 *mucA*, to promote the FW213 biofilm.

Several studies have characterized the impact of *P*. *aeruginosa* within polymicrobial communities. *Staphylococcus aureus*, a bacterium that frequently co-infects the CF airway, has been shown to enhance *P*. *aeruginosa* virulence. For instance, *P*. *aeruginosa* utilizes peptidoglycan from *S*. *aureus* to upregulate the production of extracellular virulence factors, which permits the bacterium to compete with neighboring bacteria and enhance virulence in the host [39]. *Burkholderia cenocepacia*, another common resident in CF infections, promotes *P*. *aeruginosa* biofilm development in a murine model of chronic infection [6]. Interspecies interactions with *P*. *aeruginosa* promote synergism among co-infecting bacteria to resist antimicrobials or enhance *P*. *aeruginosa* virulence. However, studies that explore the role of oral commensal streptococci in the CF lung and how they cross-talk with *P*. *aeruginosa* are currently emerging. Similar to our study, PAO1 and a clinical CF *P*. *aeruginosa* isolate do not incorporate into salivary biofilms [40]. *P*. *aeruginosa* maintains some viability planktonically during co-culture with oral bacteria, but fails to integrate into the biofilm [40]. Together, these data suggest that oral commensals possess defense mechanisms that exclude *P*. *aeruginosa* from biofilm communities in the oral cavity and CF lung.

Commensals themselves do not display virulence properties that directly induce disease, but have been implicated in heightening the virulence of pathogens. The oral commensal, *Streptococcus gordonii*, promotes the virulence of the periodontal pathogen, *Aggregatibacter actinomycetemcomitans* by producing preferred carbon sources and altering its metabolism in a manner that enhances *A*. *actinomycetemcomitans* fitness and virulence [41, 42]. Moreover, oral commensal streptococci have been shown to both attenuate and enhance virulence of the *P*. *aeruginosa* Liverpool Epidemic Strain (LES), however, the phenotypes displayed in these interactions were dependent on growth conditions and bacterial colonization sequence and did not include *S*. *parasanguinis* [43, 44]. Our previous studies have demonstrated that the oral commensal *S*. *parasanguinis* can inhibit *P*. *aeruginosa* and *A*. *actinomycetemcomitans* viability using varying mechanisms [11,12, 45]. *A*. *actinomycetemcomitans* can promote *S*. *parasanguinis* biofilm formation by modulating *S*. *parasanguinis* hydrogen peroxide production [45]. These studies signify the importance of examining more closely the role commensals have in polymicrobial infections, which will be useful in determining whether mechanisms used by commensals that interfere with the virulence of pathogens can be harnessed for the development of therapeutics. In conclusion, the ability of the oral commensal *S*. *parasanguinis* to colonize the CF environment and exploit the mucoid phenotype of *P*. *aeruginosa*, which is often associated with chronic lung infections, represents a unique bacterial interaction that could be utilized to modulate *P*. *aeruginosa* virulence.

## Materials and methods

### Bacterial strains, culture conditions and reagents

Bacterial strains are listed in Table 2. Oral streptococci were routinely grown aerobically (5% CO_2_) at 37°C in Todd-Hewitt broth (THB, Difco). *P*. *aeruginosa* was isolated on Pseudomonas Isolation Agar (PIA) and subsequently cultured in Luria-broth (LB) and incubated at 37°C. Antibiotics were purchased from Sigma-Aldrich (St. Louis, MO) and used at the following concentrations: 125 μg mL^-1^ kanamycin for *S*. *parasanguinis* and 100 μg carbenicillin ml^-1^ for *P*. *aeruginosa*. Alginate lyase was purchased from Sigma-Aldrich and alginate was purified from FRD1 based on a previously described protocol [46]. Alginate production was quantified using the carbazole method [47].

**Table 2:** Bacterial strains used in this study.

Strain	Relevant characteristics	Reference or source
SK36	*S*. *sanguinis* wild-type	[48]
DL1	*S*. *gordonii* wild-type	[49]
***S*. *parasanguinis***		
FW213	*S*. *parasanguinis* wild-type	[48]
FW213 *bapA1*	*bapA1* mutant	[19]
FW213 *fap1*	*fap1* mutant	[50]
FW213 *bapA1*-*fap1*	*bapA1*-*fap1* double mutant	[19]
FW213 *srtA*	Sortase A mutant	[17]
FW213 *srtB*	Sortase B mutant	[17]
FW213 *abpA*	*abpA* mutant	[17]
***P*. *aeruginosa***		
FRD1	CF isolate, mucoid	[51]
FRD1 *mucA*^+^	Complemented with *mucA*, non-mucoid	[38]
FRD1 *algD*	*algD* mutation, non-mucoid	[52]
FRD1 *algT*	*algT* mutation, non-mucoid	[52]
PAO1	Wound isolate, non-mucoid	[53]
PAO1 *mucA*	Deletion of *mucA* (PDO300)	[54]
CF clinical isolates	C1-C3 mucoid, C4-C6 non-mucoid	UAB CF Center

### Biofilm formation assays

Biofilm formation of *S*. *parasanguinis* and *P*. *aeruginosa* was assessed using the crystal violet staining method. The optical density of cells at 470 or 600nm was used to monitor bacterial growth. Briefly, overnight cultures of *S*. *parasanguinis and P*. *aeruginosa* were sub-cultured separately in THB or L-broth, respectively, and grown to exponential phase (A_470/600_ 0.6~0.8). Following sub-culture, *S*. *parasanguinis* cells were inoculated at a 1:1000 dilution (1×10^4^ CFU/ml) while *P*. *aeruginosa* cells were inoculated at a 1:100 dilution (1×10^5^ CFU/ml) in tryptic soy broth with 0.5% yeast extract (TSBYE) containing 1% sucrose. *S*. *parasanguinis* and *P*. *aeruginosa* were inoculated at the aforementioned ratios either separately or mixed together for mono-species or dual-species biofilm assays. Two-hundred microliters of each mixture was then added to sterile 96-well plates (Nunc) and incubated at 37°C in 5% CO_2_ for 16 h. The biofilms were stained with 0.1% crystal violet, dissolved with 30% acetic acid, and measured at 562 nm. Each assay was performed in triplicate wells and was repeated three times.

### Viability of *S*. *parasanguinis* and *P*. *aeruginosa* in co-cultures

To enumerate colony forming units (CFU), planktonic *S*. *parasanguinis* and *P*. *aeruginosa* cells from mono-species or dual-species cultures were serially diluted and plated on TSBYE agar for *S*. *parasanguinis* or PIA for *P*. *aeruginosa*. Biofilms were washed twice using phosphate buffered saline (PBS) and scraped from wells before being re-suspended, serially diluted, and plated.

### Immunofluorescence and confocal laser scanning microscopy (CLSM) analysis

Bacterial strains were grown in TSBYE in a sterile 8-well treated μ-Slide (Ibidi) under 5% CO_2_ at 37°C for 16 h. The biofilm samples were gently washed with PBS three times to remove unattached cells followed by incubation in 5% bovine serum albumin (BSA) in PBS for 1h. *S*. *parasanguinis* was incubated with a polyclonal antibody that recognizes the surface amylase binding protein (AbpA) [17] or a monoclonal antibody that recognizes the surface fimbriae protein Fap1 [20]. *P*. *aeruginosa* was incubated with a monoclonal antibody that recognizes an outer membrane protein (Omp) (Abcam). Alginate was probed using a polyclonal antibody that was provided as a gift from Dr. Gerald B. Pier at Harvard University [55]. The biofilms were washed 3 times with PBS to remove the unattached primary antibodies and then incubated with fluorescent-conjugated secondary antibodies (Molecular Probes) for 30 min. Alexa Fluor 594 (red)-conjugated goat anti-mouse IgG and Alexa Fluor 488 (green)-conjugated goat anti-rabbit IgG were used to stain bacterial cells and alginate. The stained samples were then washed 3 times prior to detection using a fluorescence (Nikon X-Cite series 120 PC) or a Nikon A1+ confocal laser scanning microscope (CLSM) (Nikon Instruments Inc.). NIS Elements microscopy imaging software was used to calculate biofilm biomass and co-localization.

### Colonization of *Drosophila melanogaster* by *S*. *parasanguinis* and *P*. *aeruginosa*

*S*. *parasanguinis* and *P*. *aeruginosa* co-infection of *Drosophila melanogaster* flies was performed as previously described [11, 12]. Briefly, *Drosophila* flies were maintained on Jazz-Mix *Drosophila* food (Fisher). Male Canton S *Drosophila* flies (1 to 3 days old) were treated with antibiotics (erythromycin, vancomycin, and ampicillin at 50 μg/ml) for 3 days and transferred to fresh food for 3 days to remove residual antibiotics. The flies were starved for 3 hours prior to being added to vials (10 flies per vial) and orally infected with bacteria. To infect flies, *S*. *parasanguinis* was grown to an A_470_ of 2.0 and 1 mL of the culture was harvested. *P*. *aeruginosa* was grown to an A_600_ of 2.0 and 1 mL of the culture was harvested. The harvested cell pellets of one or both species were re-suspended in 100 μL of sterile 5% sucrose. The re-suspended cells were spotted onto a sterile 21-mm filter paper disc (Whatman) that was placed on the surface of 5 mL of solidified 5% sucrose agar in a plastic vial (FlyBase). To determine the number of viable bacterial cells inside the flies, the surface of the flies was briefly sterilized with 70% ethanol and washed 3 times with sterile PBS. Flies were crushed with pipette tips in an Eppendorf tube containing 100 μL of saline. Serial dilutions of the homogenate were spread on PIA or TSBYE agar plates.

### Quantitative real time PCR

RNA was extracted from *Drosophila* co-infected with *P*. *aeruginosa* and *S*. *parasanguinis* co-cultures using the Direct-zol kit (Zymo Research, Irvine, CA). Residual DNA was digested using RQ1 DNase (Promega, Madison, WI). RNA was purified with the mini-RNAeasy kit (Qiagen, Venlo, Limberg), and converted into cDNA using the iScript cDNA Synthesis kit (Bio-rad, Hercules, CA). cDNA was then used for qRT-PCR with iQ SYBR Green Supermix (Bio-rad). The qRT-PCR primers used to amplify *P*. *aeruginosa* 16s rRNA and *algD* were, F-GCTGGACTATCGCCGCTG and R- ATCTCGTAACCGGTGAAGGTG, and F- GATCATCCGCAAGACCGTCG and R- TGGCAGATCACGTCCATCAC, respectively.

## Supporting information

S1 Fig Alginate lyase does not affect bacterial growth. Cultures were grown for 16 hours in TSBYE media (+/- 20U alginate lyase) and optical densities were measured with a spectrophotometer. Data are representative of three experiments performed in triplicate.
(TIF)

S2 Fig Mutations in FRD1 alginate biosynthetic genes do not promote FW213 biofilm formation. 16-hour dual-species biofilms of FW213 with FRD1, FRD1 *algD*, and FRD1 *algT* grown in 96-well plates. Biofilm biomass was measured using the crystal violet assay. Data are representative of three experiments performed in triplicate. *P<0.05 (Student’s *t*-test).
(TIF)

S3 Fig *P*. *aeruginosa* alginate production in single or dual species biofilms with FW213. Alginate production was measured from 6-hour biofilm cultures using the carbazole method. Data are representative of three experiments performed in triplicate. ns = not significant and ND = not detected.
(TIF)

S4 Fig Mucoid and non-mucoid *P*. *aeruginosa* promote FW213 growth. CFU quantification of planktonic cells of FW213 with A. FRD1 B. FRD1 *mucA*^+^ C. PAO1 and D. PAO1 *mucA*^+^. Data are representative of three experiments performed in triplicate. *P<0.05 (Student’s *t*-test).
(TIF)

S5 Fig Mucoid clinical P. *aeruginosa* isolates promote FW213 biofilm. Dual-species biofilms of FW213 with mucoid and non-mucoid *P*. *aeruginosa* clinical isolates. Biofilm biomass was measured using the crystal violet assay. Data are representative of three experiments performed in triplicate. *P<0.05 (Student’s *t*-test and ANOVA).
(TIF)

S6 Fig The effect of a FW213 *abpA* mutation on dual-species biofilm formation. 16 hour dual-species biofilm of FW213 and FW213 *abpA* with FRD1 and PAO1. Biofilm biomass was measured using the crystal violet assay. Data are representative of three experiments performed in triplicate. *P<0.05 (Student’s *t*-test).
(TIF)

S7 Fig Colonization of FRD1 and FRD1 *mucA*^+^ with FW213 and derivatives in the *Drosophila melanogaster* model. CFU quantification of FRD1 and FRD1 *mucA*^+^ recovered from *Drosophila melanogaster*. Data are representative of three experiments performed in triplicate. *P<0.05 (Student’s *t*-test).
(TIF)

S8 Fig *algD* expression in the *Drosophila melanogaster* model in single and co-infections. qRT-PCR of *algD* expression in *Drosophila melanogaster* after 24-hour infection. Data are representative of three experiments performed in triplicate. *P<0.05 (Student’s *t*-test).
(TIF)
