# Supplementary figures and images for: A commensal streptococcus hijacks a Pseudomonas aeruginosa exopolysaccharide to promote biofilm formation
Source: PLoS Pathog. 2017 Apr 27;13(4):e1006300. doi: 10.1371/journal.ppat.1006300 (PMC5407764; doi:10.1371/journal.ppat.1006300)

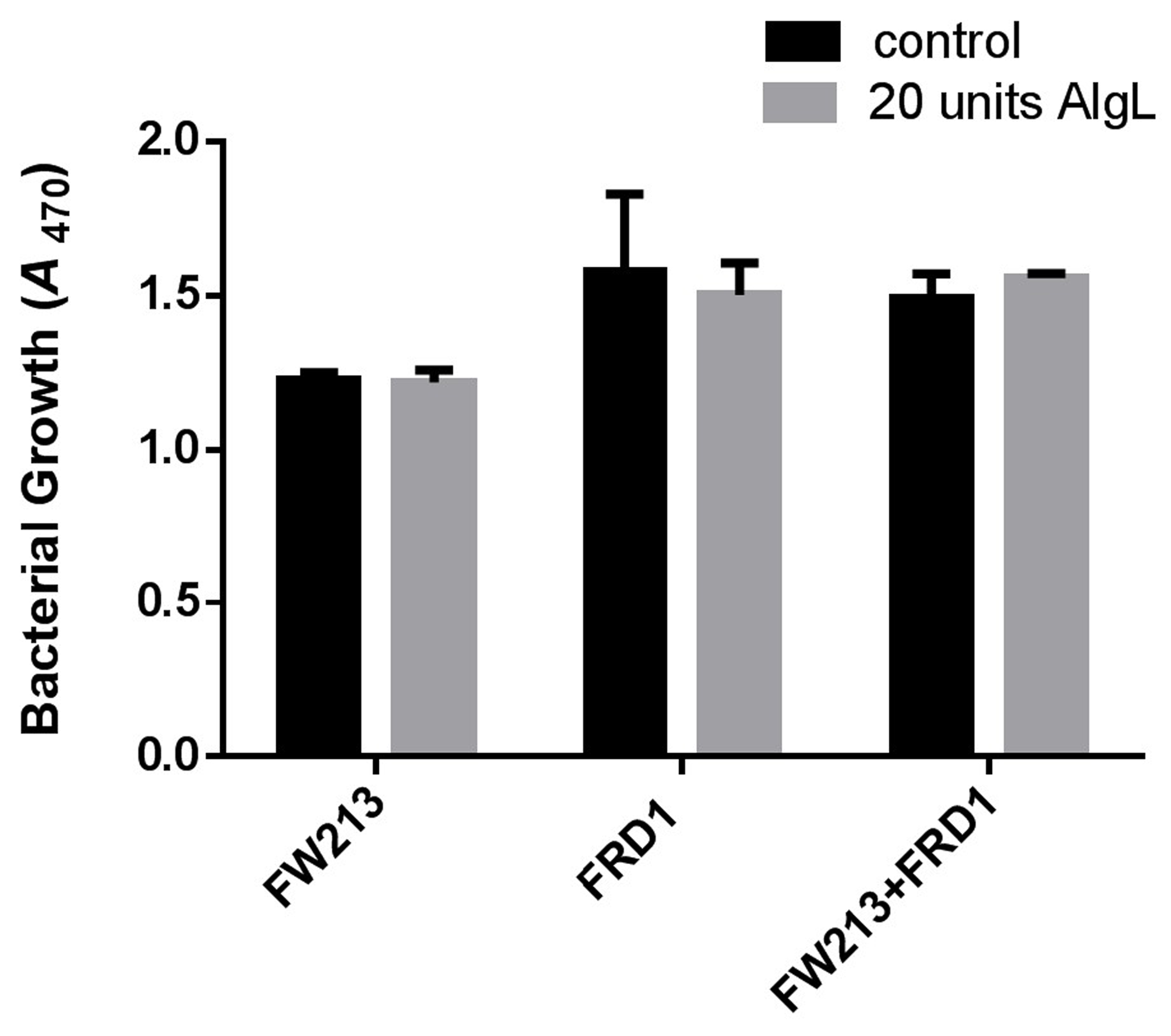

Supplement: S1 Fig — Cultures were grown for 16 hours in TSBYE media (+/- 20U alginate lyase) and optical densities were measured with a spectrophotometer. Data are representative of three experiments performed in triplicate. (TIF) [file ppat.1006300.s001.tif]

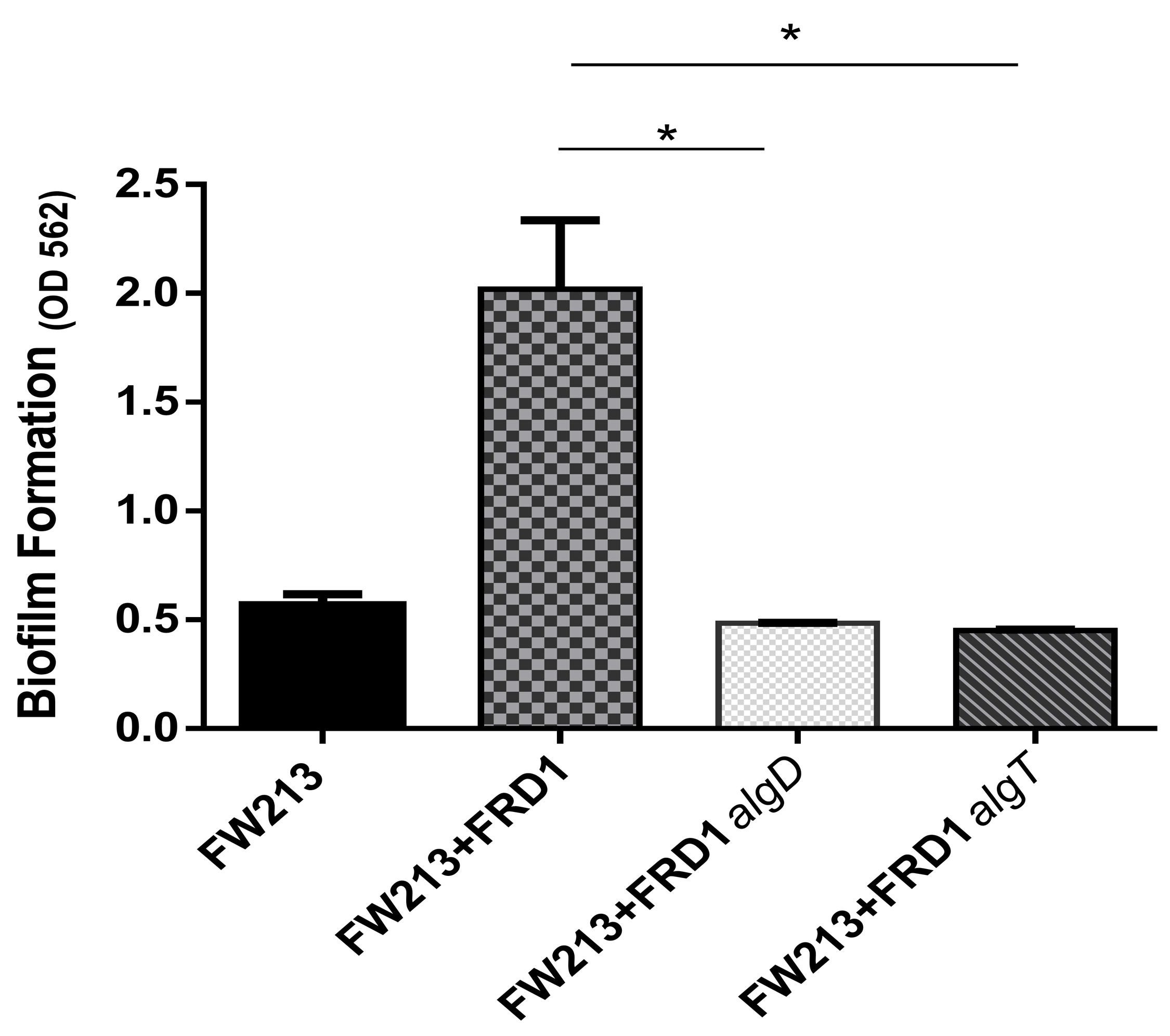

Supplement: S2 Fig — 16-hour dual-species biofilms of FW213 with FRD1, FRD1 algD, and FRD1 algT grown in 96-well plates. Biofilm biomass was measured using the crystal violet assay. Data are representative of three experiments performed in triplicate. *P<0.05 (Student’s t-test). (TIF) [file ppat.1006300.s002.tif]

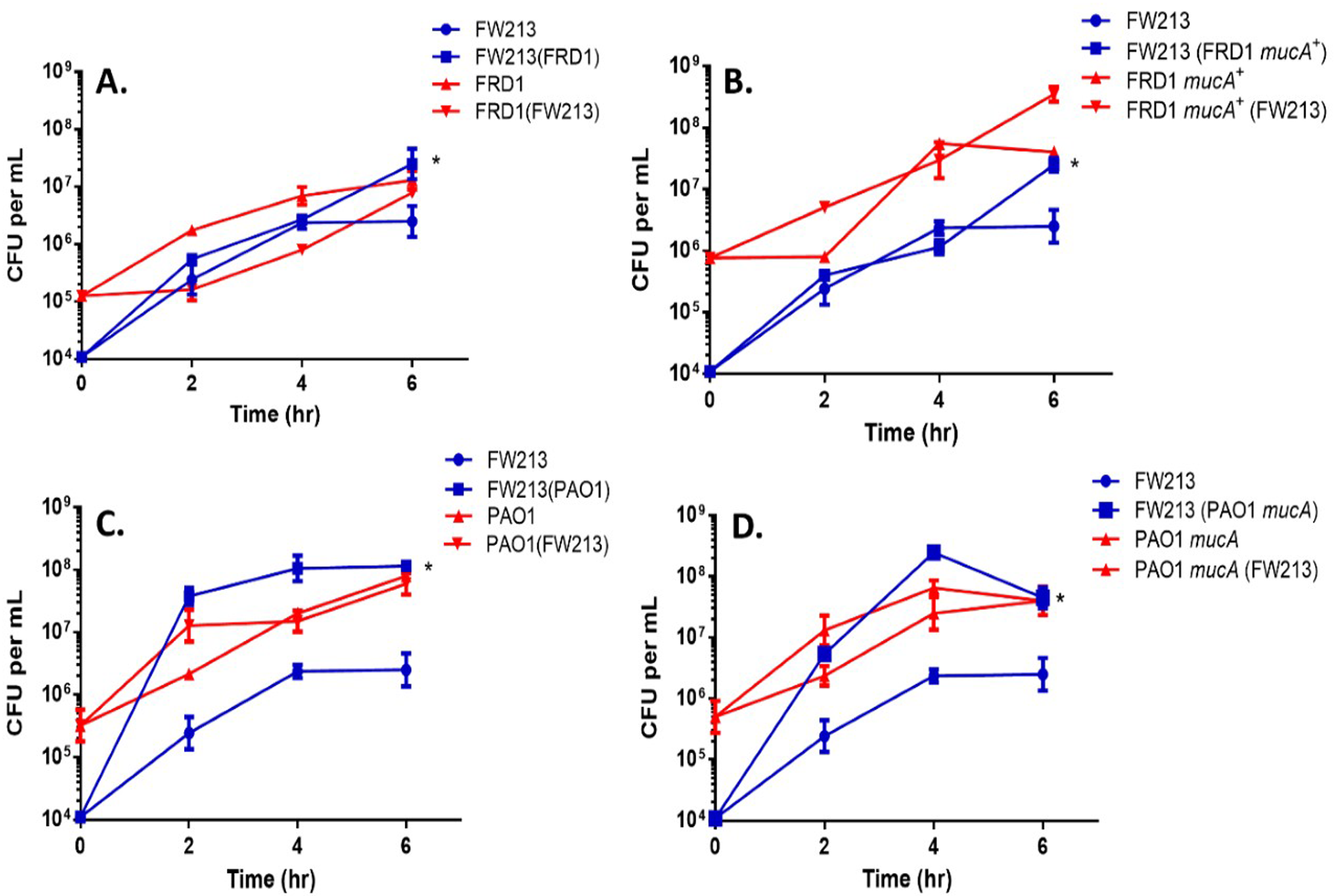

Supplement: S4 Fig — CFU quantification of planktonic cells of FW213 with A. FRD1 B. FRD1 mucA+ C. PAO1 and D. PAO1 mucA+. Data are representative of three experiments performed in triplicate. *P<0.05 (Student’s t-test). (TIF) [file ppat.1006300.s004.tif]

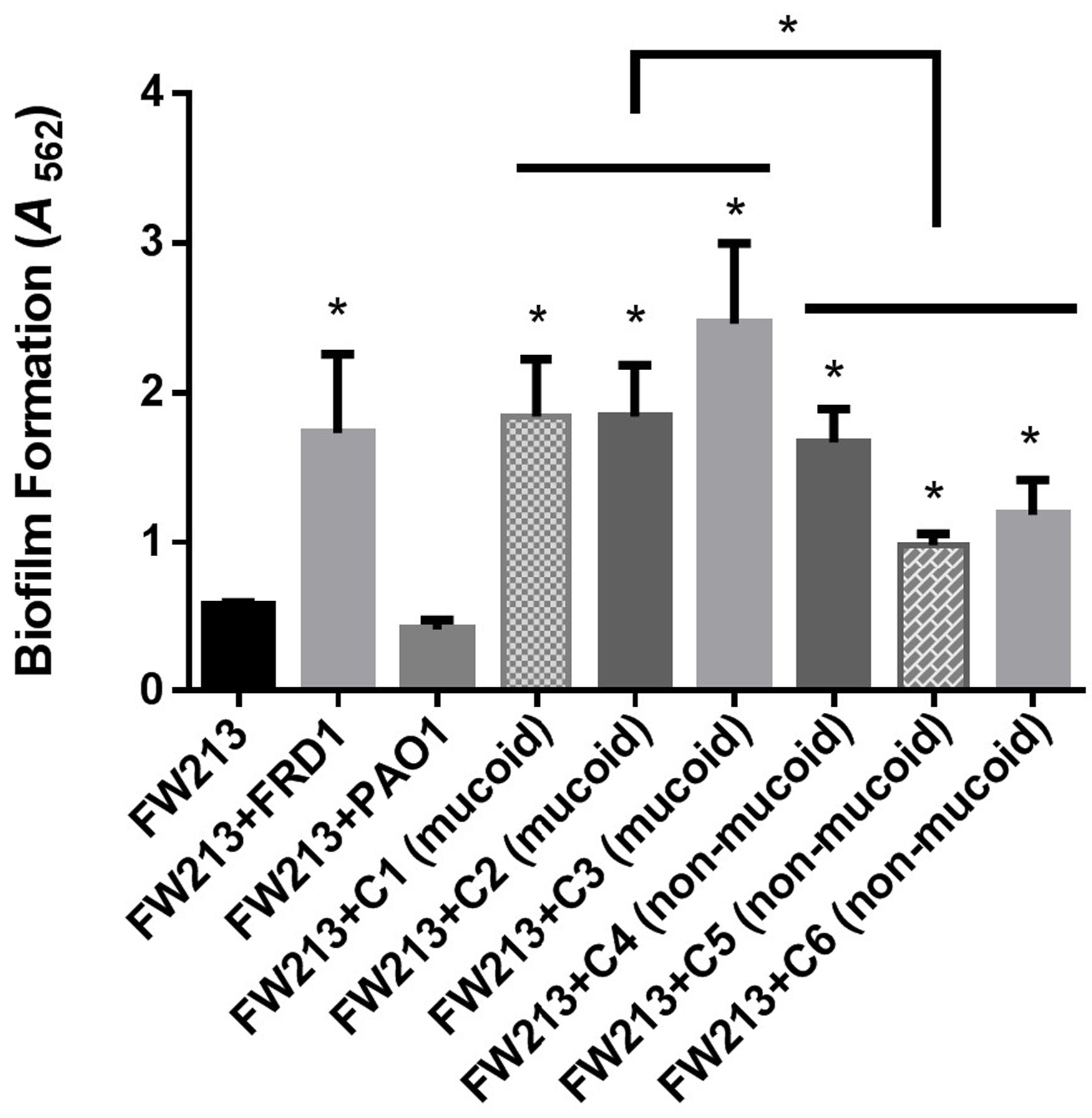

Supplement: S5 Fig — Dual-species biofilms of FW213 with mucoid and non-mucoid P. aeruginosa clinical isolates. Biofilm biomass was measured using the crystal violet assay. Data are representative of three experiments performed in triplicate. *P<0.05 (Student’s t-test and ANOVA). (TIF) [file ppat.1006300.s005.tif]

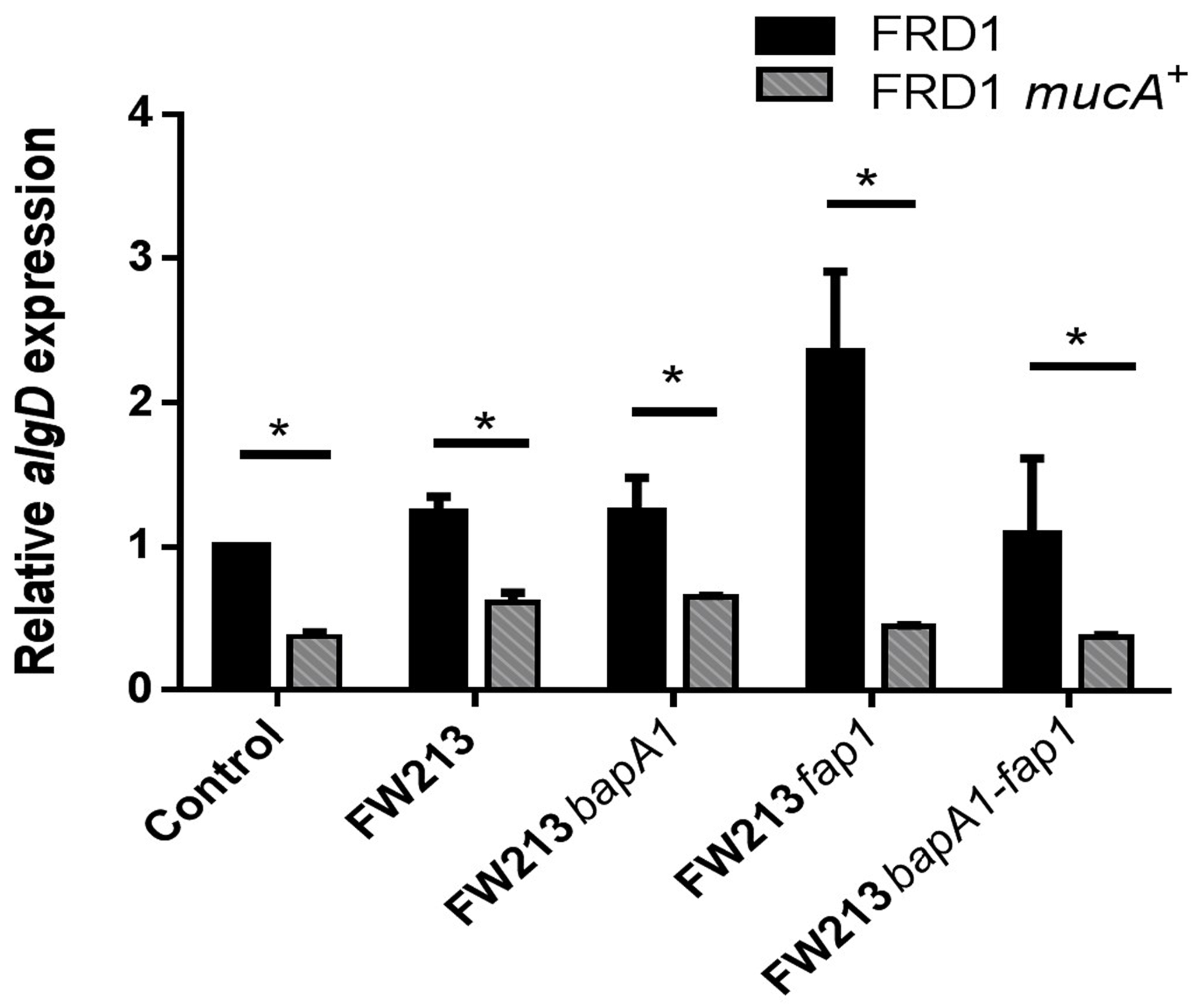

Supplement: S8 Fig — qRT-PCR of algD expression in Drosophila melanogaster after 24-hour infection. Data are representative of three experiments performed in triplicate. *P<0.05 (Student’s t-test). (TIF) [file ppat.1006300.s008.tif]
